# An assessment of infant medication administration and storage practices in selected communities in the Vhembe District of Limpopo Province, South Africa

**DOI:** 10.4102/hsag.v24i0.1075

**Published:** 2019-03-20

**Authors:** Aspen D. Flynn, Rebekah L. Scheuerle, Geoff Galgon, Stephen E. Gerrard, Vhonani O. Netshandama

**Affiliations:** 1 Department of Community Engagement, University of Venda, Thohoyandou, South Africa; 2 JustMilk Nonprofit, Laguna Beach, United States

## Abstract

**Background:**

Effective infant medication administration and storage is a major public health challenge outlined by the World Health Organization. These challenges may be exacerbated in rural or limited-resource areas.

**Aim:**

The aim of this study was to investigate infant medication administration and storage practices.

**Setting:**

This study took place in selected communities in the Vhembe District of Limpopo Province, South Africa.

**Method:**

Data was collected through 39 semi-structured interviews with infant caretakers and rural health workers. Interviews were recorded when permission was given by participants. Interviews were transcribed and coded using grounded theory and Tesch’s model of data analysis. Themes were agreed upon through consensus discussions with the researchers and an independent coder.

**Results:**

Six themes that affect current infant medication administration and storage practices in the Vhembe District were identified: access to infant healthcare, the role of health workers, the devices used in the administration of infant medication, reluctance of the infant to take the medication, storage and reuse of infant medication in the rural home and hygiene practices surrounding infant medication administration.

**Conclusions:**

Many factors were found to affect infant medication administration and storage practices in in the Vhembe District. Substantial evidence was found to suggest that the relationship between rural health workers and infant caretakers strongly influences these practices: a great amount of reliance and trust is placed in the health worker. Ensuring proper dosage of infant medication in the rural household arose as a main concern of participants. Reuse of medication in the home and home hygiene practices surrounding infant medication administration are areas of potential future research. This future research may further inform recommendations for infant medication administration and storage practices in the Vhembe District.

## Introduction

Infant medication administration and storage are major public health challenges worldwide (WHO 2011). Most infant medications are administered as a liquid using an oral syringe, dosing cup or medication spoon (Sobhani et al. 2008; Walsh, Bickmann, Breitkreutz, Chariot-Goulet & EuPFI 2011). These methods can all lead to errors in dosing (Sobhani et al. 2008; Truter, Schellack & Meyer 2017; Walsh et al. 2011). Additionally, cold chain requirements and palatability issues can affect proper storage and delivery of liquid medication (Tuleu & Breitkreutz 2013). Some infant medication is also available in the form of a solid oral dispersible tablet, which must be dispersed in clean water or milk in a clean container before being administered as above (UNICEF 2010b).

Three major causes of disease in children younger than 5 years in South Africa are HIV/AIDS, pneumonia and diarrheal diseases (WHO 2014). Treatment for these diseases in infants includes delivering a medication through one of the methods described above. HIV/AIDS treatment and prevention in infants is addressed through an antiretroviral regimen, such as a combination of abacavir, lamivudine and ritonavir-boosted lopinavir (WHO 2016). Antiretrovirals can be administered to infants in various forms including dispersible tablets, which is recommended, or as syrups via an oral syringe when tablets are not available (Havens & Gibb 2007; WHO 2010, 2016). Amoxicillin and other antibiotics are frequently used to treat pneumonia in infants and primarily take the form of dispersible tablets (Zar et al. 2005). The recommended treatment for diarrhoea includes oral rehydration salts, administered as a liquid solution via one of the administration devices described above (UNICEF 2010a).

According to the WHO, adequate instructions on drug administration are important for a caretaker to properly deliver paediatric medication (WHO 2012). Information on infant medication administration practices may be limited in rural or resource-limited areas because of contextual challenges such as limitations in supply chain, healthcare infrastructure and availability of health workers (HWs). This study assesses infant medication administration and storage practices in the Vhembe District of Limpopo, South Africa, and community factors that may influence these practices.

### Problem statement

Challenges in administering and storing infant medication in the home may be exacerbated in low-resource rural areas, where there may be limited access to resources such as healthcare, electricity, and clean water. One such region is the Vhembe District of Limpopo Province, a rural region of northern South Africa.

Limpopo is the northernmost province in South Africa and has a population of 5.4 million (Bessong et al. 2014). It is the most rural South African province, with 87% considered rural, compared to the national average of 43% (Bessong et al. 2014). The Vhembe District within Limpopo has limited access to health facilities in some areas: 49% of children in Limpopo live a distance greater than 30 min from the closest clinic (UNICEF 2011). This degree of access to health facilities may influence medication administration practices in the home. A long distance from the home to clinics, pharmacies and doctors may lead to limited dissemination of information to caregivers regarding infant medication administration. Limited electricity and frequent blackouts are common in the Vhembe District and have been cited as a challenge in the region (Tshitangoni, Okorie & Francis 2010; Uhunamure, Nethengwe & Musyoki 2017). This may decrease the local capacity to refrigerate medication, especially during the hot and rainy summer season. Moreover, weather and road conditions change frequently in the Vhembe District, especially during the rainy season, which may further exacerbate the difficulty of reaching clinics. Because many clinics are situated on dirt roads, traveling to health facilities may be a challenge. Clean water is also limited in the Vhembe District, which may affect hygiene and sanitation practices surrounding infant medication administration in the region (Majuru, Jagals & Hunter 2012; Mudau, Mukhola & Hunter 2017; Rietveld, Haarhoff & Jagals 2009).

### Purpose and objectives

This study assessed infant medication administration and storage practices in selected communities in the Vhembe District of Limpopo, South Africa. The purpose was to understand experiences in administering medication to infants and associated challenges from the perspective of infant caretakers (ICs) and rural HWs.

### Study setting

This study was conducted in the Mutale and Thulamela municipalities of the Vhembe District of Limpopo. See Table 1 for a list of major indicators in these municipalities (Statistics South Africa: Mutale 2011; Statistics South Africa: Thulamela 2011). Interviews within this region were conducted at four local government health clinics (hereafter referred to as ‘clinics’) and at participant homes in five rural communities. The clinics were Thondo Tshivhase Clinic, Rambuda Clinic, Mutale Health Centre and the University of Venda Campus Clinic, and the communities were Tshilamba, Tshishivhe, Pile, Tshapasha and Tzhagwa.

**TABLE 1:** Major indicators for study sites, the Mutale and Thulamela municipalities.

Feature	Mutale	Thulamela
**Tribal or traditional area, %**	96.8	85.4
**Mean household size**	3.8	3.9
**Female-headed households, %**	54.8	54.4
**Highest education level, %**		
No schooling	2.2	2.3
Some primary	45.5	42.7
Completed primary	7.0	6.4
Some secondary	36.7	36.0
Completed secondary	6.8	9.6
Higher education	1.1	1.9
Not applicable	0.7	1.1
**Unemployment rate, %**	48.8	43.8
**Flush toilet connected to sewerage, %**	3.8	10.7
**Piped water inside dwelling, %**	5.8	15.2
**Electricity for lighting, %**	83.3	87.2

## Research methods and design

A qualitative interpretive phenomenological design was employed. This was an explorative qualitative study, using a semi-structured interview guide developed by the researchers to collect data.

### Population and sample

The study population was comprised of ICs over 18 years of age and HWs. Participants were recruited through purposive sampling. Eligibility criteria centred on identifying with at least one of the following categories: (1) IC of a child 5 years and younger or (2) HW employed at a local healthcare institution. ICs were mothers and grandmothers who cared for an infant for more than 12 hours of the day. HWs were professional nurses, midwives and counsellors who worked in local clinics. Thirty-nine semi-structured interviews were conducted with 44 participants: 35 ICs (30 mothers and 5 grandmothers) and 9 HWs. Interviews were conducted in small groups or with individuals (see Table 2 for additional demographic information on participants).

**TABLE 2:** Participant information.

Feature	Infant caretakers		Health workers
	Mothers	Grandmothers or elders	
Number of participants	30	5	9
Mean age, years	27.9	-	46.4
Age range, years	19–45	62–84	24–60
Mean number of children	1.6	-	-
Number of children, range	1–4	-	-
**Total participants**	**-**	**44**	**-**

#### Recruitment of participants

Study sites at communities and clinics were identified through community relationships previously established by University of Venda researchers through the MAL-ED study (Bessong et al. 2014). Key informants identified participants through purposive sampling, and a snowball technique was used to identify further participants. Only those who volunteered to participate contributed to the findings of the study.

### Data collection procedure

Data collection occurred between February and October 2015. Data was collected through 39 semi-structured interviews. There were 44 participants total, as 35 interviews were individual interviews and 4 interviews with ICs were conducted in small groups of 2–3 participants. All participants provided verbal consent before each interview. Interviews were recorded when the participant gave consent for an audio recording. If the participant did not give consent for the interview to be recorded, handwritten notes were taken. Participants were asked open-ended questions about infant medication administration and storage practices using an interview guide developed by the researchers. Questions were intentionally non-specific to health areas in order to identify a broad range of experiences and reflections around infant medication administration. Interviews with ICs were conducted at participant homes in the local language of Tshivenda with an interpreter, or in English when possible. Member checking was conducted throughout the interview to ensure accurate translation. All interviews with HWs were conducted at rural health clinic offices in English. Transcription and coding of interviews began during data collection. Participant recruitment and subsequent interviews were concluded when it was determined that data saturation was reached.

### Data analysis

Data was analysed through a constant comparison analysis method within and between interviews of different participants (Strauss & Corbin 1990). Interviews were transcribed and coded into themes and subcategories, which were verified with an independent coder through a consensus discussion. Data analysis was modelled on Tesch’s methods of qualitative data analysis (Tesch 1990). Member checking was conducted with selected participants.

### Ethical considerations

Ethical approval for the study was granted by the University of Venda Research Ethics Committee (CE/14/CE/01/1605), the Vhembe District Municipality Department of Health and Social Development as well as the Limpopo Department of Health.

### Trustworthiness

All four aspects of trustworthiness were applied to this study (Lincoln & Guba 1985). To ensure credibility, the lead researcher (who is also first author) spent a full year in the Vhembe district. This prolonged engagement allowed the researcher to establish a positive relationship with the community and become familiarized with the study setting. To enhance credibility, researchers interacted with participants at least three times – to establish relationships, conduct the interviews or focus groups, and perform member-checking. Triangulation of findings was established through semi-structured interviews, focus group discussions, and observation. All findings were confirmed by an independent coder. To ensure transferability, a detailed description of the study setting, as well as the research methodology, is provided. This will allow future researchers to determine if these findings can be justifiability applied to other settings. The description of the research methodology and the comprehensive description of the background of the current research ensures dependability, allowing a future researcher to repeat the study.

Records of the research path and all notes and interview transcripts have been saved to enhance confirmability. The substantial quotes present as a part of this manuscript also ensure confirmability. The principal investigator of this study is local to the study setting and a professor at the region’s university. The first author, while not originally from the study setting, spent one year in the region and worked under the direct mentorship of the principal investigator.

## Results

Six major themes emerged surrounding infant medication administration and storage practices in the Vhembe District. The themes were access to infant healthcare, the role of HWs, the devices used in the administration of infant medication, reluctance of the infant to take the medication, storage and reuse of infant medication in the rural home, and hygiene practices surrounding infant medication administration.

### Access to infant healthcare

Most ICs reported that when infants become sick, they first take them to the nearest clinic by walking or by taking a local minibus taxi. Some ICs expressed difficulty in getting to the clinic because of the distance or because they could not afford the taxi fare.

‘It is a long distance … Two hours. Two hours … It is too far. There is not a clinic in our village.’ (P11, Mother, 32 years)

Some ICs stated that they often had to wait several hours before the infant was seen at the clinic.

‘Hey, when we go there at clinic we … We find a lot of people at – at that time, even though there is no people, they don’t attend us. They just sit and … and just make like they didn’t see us … We can spend more than an hour there at clinic … Just waiting.’ (P20, Mother, 37 years)

At the four clinics that participated in this study, the highest trained staff were often nurses, with doctors present a couple of hours per week. Many ICs cited the need to go to an additional government clinic, private practitioner, hospital or pharmacy before they could access the medication or clinical expertise needed to appropriately treat their infant. This was stated to be a time- and cost-intensive process, as these facilities were at a greater distance from the community.

‘But, as a mother, I feel bad about the … about bad happening to my son. So … I think that … the clinic doesn’t do anything. So that’s why I take [*him*] to the doctor and the doctor do something about that.’ (P32, Mother, 21 years)

### Role of health workers

Interviews revealed that clinic staff play a significant role in influencing infant medication administration practices.

‘I believe everything that the nurses say.’ (P2, Grandmother, 62 years)

All HWs interviewed stated that they always gave instructions to an IC on how to deliver the prescribed infant medication and often asked the IC to repeat these instructions during the clinic visit. Many HWs also wrote medication delivery instructions on the medicine bottle in the form of simple symbols, as not all ICs are fully literate. Some ICs mentioned that medication administration is also performed by grandmothers, implying that the individual receiving instructions from the nurse may not be the same individual who delivers the medication.

Many HWs expressed doubt that all ICs are able to properly deliver medication in the home. There was particular scepticism directed towards the ability and willingness of grandmothers to accurately measure and administer liquid medication and follow proper hygiene practices.

‘… some of the infants, they are left with their grannies … you can tell the grannies that this baby must take this medication … when she’s come back for second visit, when you are questioning, you find that there is missing doses. She’s not checking as she was being told last time. And you end up learning sometimes those babies are not taking medication, correct dose.’ (P39, HW, 24 years)

HWs occasionally expressed frustration at the difficulty in following up with ICs to ensure correct medication administration practices.

‘I do not have anything that I can do to ensure that all the medication that is intended to be given to a child is given.’ (P37, Nurse, 43 years)

In contrast to this doubt expressed by HWs, most ICs stated that they understood the HW’s instructions and were able to carry them out in the home to the best of their abilities.

‘If somebody tells me the instruction, I will think about it when using the medicine.’ (P32, Mother, 21 years)

### Infant medication administration devices

Oral syringes, dosing cups and spoons were the common methods of delivering liquid medication to an infant in the Vhembe District. Most HWs stated that each of these delivery devices were available in the clinic. However, probing (follow-up questions during interviews) revealed that oral syringes were only used with newborn babies and were only available in hospitals, with the exception of antiretrovirals, which were packaged with an oral syringe and more widely available.

Most ICs reported that the only delivery device a clinic provided was a plastic spoon. ICs revealed that the most common practice of delivering medication to an infant in the home was pouring liquid medicine into either a plastic spoon provided by the clinic, a household teaspoon (conventionally used to put sugar in tea) or the lid of the medication bottle and subsequently pouring the medication into the infant’s mouth. The majority of ICs cited instances where the clinic did not provide a delivery device and instead recommended the use of a household teaspoon. The frequent use of these alternative methods of delivering medications suggests discrepancies in measuring the proper dose, as these household teaspoons differ in size from the clinic spoon and from each other and lack the appropriate markings to ensure accurate dosage.

‘Sometimes they give us the spoon from the clinic. Sometimes we find there is no spoon. They will say you must use your own teaspoon at home … It’s different. Different. Because spoon at home is a steel. And spoon at hospital is a plastic one … I just pour.’ (P11, Mother, 32 years)

Some ICs expressed difficulty or uncertainty in measuring liquid medication, even with the spoon provided by the clinic.

‘To measure it’s difficult. You can’t measure right sometimes …’ (P29, Mother, 38 years)

When ICs were asked which device they felt most comfortable using, many indicated an oral syringe, even if they had limited to no experience using it, with the reason being that it provided greater ease for both medication measurement and administration. Both HWs and ICs mentioned that when using any administration device, it is often difficult to determine if the infant has swallowed the entire liquid medication. Participants frequently stated that a portion of the medication often drips down the side of the infant’s mouth rather than the infant swallowing the entire dose.

### Reluctance of infant to take medication

Both ICs and HWs stated that many babies do not like the taste of liquid medications and acknowledged the difficulty of delivering medication to an infant.

‘It’s always a struggle because they spit it out.’ (P2, Grandmother, 62 years)

ICs often mentioned squeezing the mouth of the infant to encourage swallowing and holding the hands of the infant so he or she does not hit the medication spoon away before it enters the mouth.

‘By himself he can’t swallow the medicine so I supposed to squeeze the mouth. Then the medicine will go through … It is not easy because sometimes she – he will be refusing to swallow.’ (P6, Mother, 24 years)

Several ICs described the need to ‘force’ the infant to swallow the medication, either through squeezing the mouth or through other means. This was confirmed through observation of mothers delivering medication to their infants.

‘She doesn’t like medicine so I forced her. That’s why I do that thing of squeezing the mouth. I was forcing it.’ (P34, Mother, 30 years)

Participants communicated a sense of frustration regarding their awareness of the need for the infants to take medication and the reluctance of the infant to swallow the medication.

### Storage and reuse of infant medication in the rural home

Medication was stored primarily in the refrigerator or in a typical cupboard called a ‘room divider’ present in most rural homes.

Most ICs expressed familiarity with the concept of medicine expiry dates, and many disposed of the remaining medication after the infant’s condition improved. Some, however, kept the bottle to use the medication again at a later date either with the same infant or another child in the family.

‘I don’t throw them. When they become sick again I just give her it again.’ (P23, Mother, 29 years)

One grandmother mentioned that many ICs know that this is an incorrect and potentially dangerous practice, but it is common nonetheless.

‘We do it knowing it, that it’s not correct, but it’s like we are wasting.’ (IC, Grandmother, 65 years)

### Hygiene practices surrounding infant medication administration

Most ICs reported that they cleaned the medication administration device either after use or before the next use. Participants often stated this as important because they did not want the delivery device to be contaminated.

‘I am scared of the germs.’ (P11, Mother, 32 years)

Household water sources varied: a tap within a household plot, a community tap and a nearby river were all stated as sources of water used for cleaning.

## Discussion

It is important for clinic staff to properly educate patients and families on appropriate infant medication administration practices when they have an opportunity, as patient visits were stated as limited. Long wait times at clinics and the frequent need to travel to additional health facilities are both burdens to ICs. Waiting times encountered by patients and ICs may be partially because of a long line of patients or work overload at the clinic. These factors may also affect the lack of HW follow-up with ICs regarding infant medication delivery in the home. More research on the causes of these issues is encouraged.

The researchers observed some scepticism held by HWs about the ability of the ICs, especially grandmothers, to properly administer medication to an infant. This mistrust appeared related to the common practice of multiple individuals caring for one infant in the household. Because the individual administering medication to an infant in the home may be different than the individual who brought the infant to the clinic and received instruction, medication administration instructions may need to be transferred from one IC to another within the household. There may be potential for distortion of information in this information transfer process, potentially affecting proper medication delivery. Further studies are warranted to more thoroughly understand the relationship between the HW and the IC.

Common methods of administering medication to an infant using a clinic spoon or home teaspoon may lead to ambiguous and inconsistent dosage of liquid medication. Frequent shortages of clinic spoons in the region may exacerbate this inconsistency in dosage by necessitating the use of household teaspoons, which differ in size. Reluctance of the infant to swallow the liquid medication and the subsequent need to force the infant to swallow may also influence dosing accuracy, as well as cause frustration for the IC. IC preference for an oral syringe, even with little to no experience in its use, indicates an interest in devices that may deliver infant medication more easily and effectively. Additional research on dosage accuracy and potential effects on the health of infants is encouraged.

Recent World Health Organization principles advise the use of infant antiretrovirals (ARVs) in the form of dispersible tablets. All infant ARVs described by participants and observed by the researchers were in syrup form and administered via an oral syringe. Additional research on infant ARV administration in the region may be warranted.

Comments describing medication reuse, particularly without regard for the expiry date, suggest that more research into this practice may be beneficial to ensure infant health in the household. While few obstacles regarding cleaning infant medication administration devices were mentioned, the potential effects of water quality and limited water availability in the study region may influence proper hygiene practices (Majuru et al. 2012; Rietveld et al. 2009). Additional research on how water quality and availability may affect device use, cleanliness and hygiene in rural households in the Vhembe District is suggested.

## Study limitations

On-site interpretation of many interviews with ICs resulted in transcription and coding only after responses had been translated from Tshivenda to English. While the interpreter was fluent in both languages, some nuances of responses may have been lost in translation or misinterpreted during data analysis.

As all interviews were conducted on-site, interview conditions were not standardised across all participants. However, this same interview strategy decreased the likelihood of making the participant uncomfortable, as interviews occurred in the homes, communities and offices of the participants.

## Significance of work

This research highlights significant factors that may affect infant medication administration and storage in the Vhembe District, especially in the rural household. It also considers the importance of the relationship between the rural HW and the IC, a relationship that extends into all realms of health in rural areas. An additional implication from this research is the need to consider innovative ways in which the administration of correct dosage of infant medication could be improved or made less challenging in rural households.

## Conclusion

A qualitative study investigating infant medication administration and storage practices was conducted in selected communities in the Vhembe District of Limpopo, South Africa. It was concluded that many factors affect proper infant medication administration practices in rural areas in this district. Six main themes that arose during interviews were access to health clinics, the role of the HWs, reluctance of the infant to take medication, the devices used in the administration of infant medication, storage and reuse of infant medication in the rural home and hygiene practices surrounding infant medication administration.

Challenges with properly dosing infant medication was the most heavily emphasised topic by both ICs and HWs. Inconsistencies in infant medication administration devices is common in the Vhembe District and may influence proper dosing practices.

Additional research is warranted around factors affecting the relationship of the IC and the HW, the effects of variations in medication dosing on infant health in the region and the effects of water availability and quality on infant medication delivery and overall lifestyle in the rural home. Studies in these areas will provide greater insight into the extent to which these factors affect infant medication administration in the Vhembe District.
